# U.S. DOE Progress Towards Developing Low-Cost, High Performance, Durable Polymer Electrolyte Membranes for Fuel Cell Applications

**DOI:** 10.3390/membranes2040855

**Published:** 2012-12-18

**Authors:** Cassidy Houchins, Greg J. Kleen, Jacob S. Spendelow, John Kopasz, David Peterson, Nancy L. Garland, Donna Lee Ho, Jason Marcinkoski, Kathi Epping Martin, Reginald Tyler, Dimitrios C. Papageorgopoulos

**Affiliations:** 1 SRA International, Inc., Fairfax, VA 22033, USA; E-Mail: cassidy.houchins@ee.doe.gov; 2 U.S. Department of Energy, Washington, DC 20585, USA; E-Mails: gregory.kleen@go.doe.gov (G.J.K.); david.peterson@go.doe.gov (D.P.); nancy.garland@ee.doe.gov (N.L.G.); donna.ho@ee.doe.gov (D.L.H.); jason.marcinkoski@ee.doe.gov (J.M.); kathi.epping@ee.doe.gov (K.E.M.); reginald.tyler@go.doe.gov (R.T.); 3 Los Alamos National Lab, Los Alamos, NM 87545, USA; E-Mail: jacob.spendelow@ee.doe.gov; 4 Argonne National Lab, Lemont, IL 60439, USA; E-Mail: kopasz@anl.gov

**Keywords:** polymer electrolyte membranes, fuel cells, proton exchange electrolytes, PEMFC, direct methanol fuel cells, DMFC

## Abstract

Low cost, durable, and selective membranes with high ionic conductivity are a priority need for wide-spread adoption of polymer electrolyte membrane fuel cells (PEMFCs) and direct methanol fuel cells (DMFCs). Electrolyte membranes are a major cost component of PEMFC stacks at low production volumes. PEMFC membranes also impose limitations on fuel cell system operating conditions that add system complexity and cost. Reactant gas and fuel permeation through the membrane leads to decreased fuel cell performance, loss of efficiency, and reduced durability in both PEMFCs and DMFCs. To address these challenges, the U.S. Department of Energy (DOE) Fuel Cell Technologies Program, in the Office of Energy Efficiency and Renewable Energy, supports research and development aimed at improving ion exchange membranes for fuel cells. For PEMFCs, efforts are primarily focused on developing materials for higher temperature operation (up to 120 °C) in automotive applications. For DMFCs, efforts are focused on developing membranes with reduced methanol permeability. In this paper, the recently revised DOE membrane targets, strategies, and highlights of DOE-funded projects to develop new, inexpensive membranes that have good performance in hot and dry conditions (PEMFC) and that reduce methanol crossover (DMFC) will be discussed.

## 1. Introduction

In 2010, transportation accounted for 28% of the total energy used in the United States, and 93% of the fuel used for transportation came from petroleum [1]. According to the U.S. Energy Information Administration, 83% of the energy used in the United States comes from fossil fuel sources [2]. Fuel cells are part of a portfolio of technologies being supported by the U.S. Department of Energy (DOE) to reduce petroleum consumption in the United States. Fuel cells efficiently convert chemical to electrical energy and can operate on clean, domestically produced and renewable fuels. Polymer electrolyte membrane fuel cells (PEMFCs) directly fueled by hydrogen are well-suited to applications, such as light-duty vehicles and back-up power, that require fast start up times. Direct methanol fuel cells (DMFC) are mainly being developed for portable power applications. 

Transportation represents one of the more challenging applications for fuel cells, because the cost and performance requirements for fuel cells to compete with internal combustion engines (ICEs) are more demanding than those for existing fuel cell applications, such as back-up power and material handling. To be competitive in fuel cell electric vehicles (FCEVs), fuel cells must be able to match or outperform ICEs in life-cycle cost, durability, performance, and reliability. In addition, FCEVs should be able to operate under realistic environmental conditions (−40 °C to 40 °C, with typical roadside contaminants). In order to compete with ICE vehicles on cost, durability and performance, the DOE has set system level fuel cell targets and supports efforts to develop a 60% efficient (at 25% of rated power), 5000 h durable, direct hydrogen fuel cell power system for transportation applications at a cost of $30/kW [3]. System level targets are currently under review.

**Figure 1:**
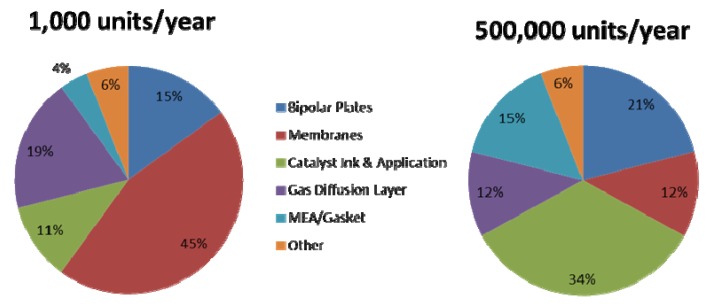
Comparison of the fractional contribution to the fuel cell stack at production rates of 1000 and 500,000 units per year [4]. At low production volumes, membrane costs account for as much as 45% of the total cost of the stack.

To achieve fuel cell system level targets, the DOE funds a number of R&D projects which address cost, durability, and performance of fuel cell components. The DOE has targeted membrane R&D as one critical component of the strategy to improve PEMFC cost and durability. A recent cost analysis of automotive fuel cells projected membrane cost to contribute as much as 45% of the total cost of the fuel cell stack for FCEVs at low production volumes (1000 systems per year) critical for near-term market penetration, with a lower contribution at high production volumes (500,000 systems per year), as shown in Figure 1 [4,5]. The analysis was based on a Nafion^®^ ionomer coating and filling the pores of a highly porous expanded polytetrafluoroethylene (ePTFE) substrate. The analysis was extended to stationary PEM fuel cell systems for prime and backup power operating on natural gas and it was found that, while the membrane costs are also high, the fuel reformer is a major cost component for stationary PEMFC systems [6]. While reductions in membrane cost would lower the cost of the fuel cell system for stationary applications, transportation applications would be the main beneficiary.

Commercial membrane polymers for PEMFCs are usually composed of a poly(tetrafluoroethylene) backbone with attached regular side chains terminating in sulfonic functional groups. Nafion^®^ (DuPont), containing sulfonic acid groups attached to perfluorinated side chains, is widely used because it offers good chemical and thermal stability with a proton conductivity of around 0.1 S/cm when fully hydrated at 30 °C [7]. However, the conductivity falls when the level of hydration in the membrane is reduced. As water is required to transport protons from the anode to the cathode, the performance of PEMFC systems with perfluorosulfonic acid (PFSA) membranes rapidly deteriorates with decreasing relative humidity (RH) as water is lost from the membrane [8]. At the other extreme, the cathode can become flooded if the humidity is too high resulting in performance loss. Maintaining the optimal hydration of the membrane requires additional water management (air and fuel humidifiers, sensors, water re-circulators, *etc.*) adding cost and complexity to the fuel cell system. The conductivity of PFSA membranes drops as temperature increases due to drying of the membrane, increasing the need for complex water management for high temperature operation. Operating at temperatures below 80 °C requires larger radiators than for ICE vehicles to maintain adequate heat rejection.

DOE has identified key areas where membrane performance must be improved for PEMFCs; these include expanding the temperature range up to 120 °C and lowering the humidification requirements of the stack. As discussed above, lowering the humidification requirements and increasing the operating temperature will decrease the cost and complexity of the fuel cell system by allowing the water and thermal management systems to be simplified or eliminated altogether. In addition to reducing the cost and complexity, fuel cell performance is improved due to faster oxygen reduction reaction kinetics at higher temperatures, allowing use of a smaller, less-expensive stack. Furthermore, the susceptibility of Pt-based catalysts to fuel contaminants such as CO decreases at higher temperatures, relaxing the need for the highest quality fuel. Finally, membranes must be made using materials and processes that can be cost-effectively scaled to commercial volumes. New low-cost membranes, which perform as well as or better than Nafion^®^, but at elevated temperatures and low RH, must be developed.

Adequate water management is also an issue for direct methanol fuel cells (DMFCs), and proper membrane water management again can allow for simplification of the balance of plant. Lower RH operation is of little concern in the DMFC, where the anode electrode is supplied with large amounts of liquid water from the fuel. Indeed, the membrane’s high affinity for water at lower temperatures is problematic in the DMFC, since the water concentration gradient between the electrodes in the membrane electrode assembly (MEA) leads to significant amounts of water at the cathode. By controlling membrane properties water transport back to the anode can be enhanced to eliminate water collection at the cathode and to eliminate active pumping back to the anode. An additional challenge to DMFCs using PFSA membranes is performance loss due to methanol crossover from the anode to the cathode through the membrane. Therefore, DMFC membranes must be developed with reduced methanol permeability.

To address the challenges facing fuel cell membranes, the U.S. Department of Energy (DOE) Fuel Cell Technologies Program (the Program) supports efforts to develop inexpensive, durable, and high performing membranes. In this paper, we review the recently revised targets and highlight DOE’s approaches and recent progress achieved in advancing membranes for fuel cell applications.

## 2. DOE Targets

The Program sets market-driven cost and technical targets to guide and prioritize R&D efforts and to measure progress towards achieving the goal of overcoming the technical and economic barriers to fuel cell commercialization. With input from the U.S. DRIVE Partnership, which includes automotive and energy companies, the DOE has identified cost and performance thresholds for PEMFC systems to be competitive with ICEs. The U.S. DRIVE partnership comprises the U.S. Department of Energy; U.S. Council for Automotive Research, representing Chrysler Group LLC, Ford Motor Company and General Motors; Tesla Motors; five energy companies—BP America, Chevron Corporation, ConocoPhillips, ExxonMobil Corporation, and Shell Oil Products US; two utilities—Southern California Edison and Michigan-based DTE Energy; and the Electric Power Research Institute (EPRI). U.S. DRIVE’s mission is to accelerate the development of pre-competitive and innovative technologies to enable a full range of affordable and clean advanced light-duty vehicles, as well as related energy infrastructure. The system level targets for cost, performance, and durability for an integrated transportation fuel cell power system operating on direct hydrogen are $30/kW, 60% electrical efficiency at 25% rated power, and 5000 h durability, respectively. At the stack component level, DOE sets targets for membranes, catalysts, membrane-electrode assemblies, and bipolar plates as needed to meet the system level targets. The DOE membrane targets, which have been recently revised [3], are summarized in Table 1. Membrane targets reflect the fact that membranes are expected to fulfill a number of roles in a PEMFC. The membrane must act as a barrier to gas transport and as a mechanical separator between anode and cathode; it must act as an ionic conductor and an electronic insulator; it must be made using materials and processes that are inexpensive; and it must last for thousands of hours in a corrosive environment containing oxygen, peroxides and Pt-based catalysts.

**Table 1:** The U.S. Department of Energy (DOE) membrane targets for an 80 kWe (net) integrated transportation fuel cell power system operating on direct hydrogen [3].

Characteristic	Units	2011 Status ^a^	2017 Targets	2020 Targets
Maximum oxygen crossover ^b^	mA/cm^2^	<1	2	2
Maximum hydrogen crossover ^b^	mA/cm^2^	<1.8	2	2
Area specific proton resistance at:				
120 °C and water partial pressures from 40-80 kPa	Ohm cm^2^	0.023 (40 kPa)	0.02	0.02
		0.012 (80 kPa)		
80 °C and water partial pressures from 25-45 kPa	Ohm cm^2^	0.017 (25 kPa)	0.02	0.02
		0.006 (44 kPa)		
30 °C and water partial pressures up to 4 kPa	Ohm cm^2^	0.02 (3.8 kPa)	0.03	0.03
−20 °C	Ohm cm^2^	0.1	0.2	0.2
Operating temperature	°C	<120	≤120	≤120
Minimum electrical resistance	Ohm cm^2^	−	1000	1000
Cost ^c^	$/m^2^	−	20	20
Durability ^d^:				
Mechanical	Cycles with <10 sccm crossover	>20,000	20,000	20,000
Chemical	hours	>2300	>500	>500

^a ^Status represents PFIA membrane as described in [9]; ^b ^Tested in MEA at 1 atm O_2_ or H_2_ at nominal stack operating temperature, humidified gases at 0.5 V DC; ^c ^Costs projected to high-volume production (500,000 stacks per year); ^d ^Based on U.S. DRIVE Fuel Cell Tech Team Cell Component Accelerated Stress Test as described in [10].

Fuel and oxidant crossover through the membrane results in reduced fuel cell performance through the formation of mixed electrode potentials, as well as through reduced fuel utilization. The hydrogen crossover target allows a nominal loss of <1% current from hydrogen crossover at 300 mA/cm^2^ and lower still at 1000 mA/cm^2^. Similarly, the membrane must provide electrical resistance to prevent shorting of the fuel cell. Under the conditions listed in footnote d of Table 1, the minimum electrical resistance target sets a nominal limit of approximately 0.3% current loss at 300 mA/cm^2^ due to electronic conduction through the membrane.

Membrane targets specify area specific resistance (ASR) instead of ionic conductivity, which allows for greater flexibility in approaches to improve membrane performance through reduction in membrane thickness. The Program has set a target of 0.02 Ohm cm^2^ at a water vapor pressure of 40–80 kPa, which is equivalent to a conductivity of 0.1 S/cm in a 20 µm membrane at 120 °C and 20%–40% RH. The target must be met over the entire range of humidity. Prior to the release of the targets described in Table 1, DOE-funded high temperature membrane projects were required to achieve a conductivity target of 0.1 S/cm at 120 °C and 25%–50% RH. Many of the projects discussed in Section 3 report the ionic conductivity instead of ASR.

Membrane costs can contribute significantly to the fuel cell stack cost. Strategies to achieve the DOE cost targets include development of manufacturing processes that are scalable and start from inexpensive precursors. One of the cost drivers for PFSA is the complexity of processing tetrafluoroethylene [11]. Motivated to bring the cost down for membranes, some projects have focused on hydrocarbon membranes which do not have tetrafluoroethylene (TFE) as a starting material. Another way to reduce system cost is through operation at higher temperature and lower relative humidity, which would off-set membrane costs through the use of simpler fuel cell systems with smaller radiators for heat rejection and with no inlet humidification.

Membranes must be durable for thousands of hours, over thousands of start-stop and humidity cycles, in a corrosive environment. For PFSA, a primary chemical degradation mechanism involves an unzipping mechanism via radical oxidation of the carboxylic acid end-group by hydrogen peroxide, which may also include cleavage of the C–O or C–S bonds in the side-chain [12]. Hydrogen peroxide can be formed during the oxygen reduction reaction (ORR) at the cathode from a 2-electron reduction pathway, as well as from 2-electron reduction of oxygen on metal particles in the membrane or on the anode. Membranes that are stable to peroxide attack and engineering strategies that reduce peroxide formation are two approaches being explored to reduce membrane degradation [13,14]. Chemical durability testing is performed according to the protocols in Reference [10] and is targeted for >500 h. Mechanical durability must also be addressed. Cracks and pinholes result in increased fuel/oxidant crossover, which reduces performance and accelerates degradation. Modeling at Lawrence Berkeley National Laboratory shows that cycling of Nafion between high and low RH promotes void growth [15]. Mechanical degradation can be mitigated through the use of high tensile strength materials with low in-plane swelling.

In order to establish a common approach for predicting and measuring the durability of PEMFC components under simulated automotive drive cycle conditions, the US DRIVE Fuel Cell Tech Team has published a collection of accelerated stress tests and recommended conditions for measurement of polarization curves [10]. The specific conditions and cycles in the protocols are intended to isolate effects and failure modes, and are based on assumed, but widely accepted, degradation mechanisms. For membranes and membrane electrode assemblies, chemical degradation is distinguished from mechanical degradation. Chemical degradation is monitored by the evolution of fluoride ions, hydrogen crossover, loss in open-circuit voltage, and shorting resistance under steady-state open circuit voltage. Mechanical durability is monitored by hydrogen crossover and shorting resistance under relative humidity cycling. Finally, standard methodologies for in-plane and through-plane membrane conductivity measurements have been developed at the Florida Solar Energy Center under a project with DOE [16].

Projects supported by the DOE have successfully developed membranes that address the major technical challenges posed by the targets. This point is illustrated in Table 1 by the fact that all but two of the 2017 targets have been achieved. The ASR status at 120 °C and 40 kPa is 0.023 Ohm cm^2^ *vs.* the target of 0.02 Ohm cm^2^, which is within 15% of the target. The cost status remains to be rigorously evaluated for high temperature membranes in the way costs have been evaluated for PFSA membranes. The ultimate goal is for all targets to be accomplished simultaneously. Specific strategies currently being explored for improving membrane performance and durability are discussed in the next section in detail. 

## 3. Technical Approach and Accomplishments

### 3.1. High Temperature Membranes for Automotive PEMFC

As has been noted previously [16,17,18] most DOE-supported work on high-temperature membranes for automotive applications has focused on the use of low equivalent weight ionomers, which are known to have higher conductivity under dry conditions than ionomers typically used in fuel cells. Developing novel membrane chemistry has therefore been an important aspect of several projects. Furthermore, the need to simultaneously achieve ASR, chemical stability, mechanical strength, durability, and cost targets poses a significant challenge. Improvements in one characteristic can often negatively impact another characteristic. A prime example is low equivalent weight PFSAs that have excellent proton conductivities, but suffer from poor mechanical durability due to increased swelling [17]. The High Temperature Membrane Working Group (HTMWG) was established between industry, academia and national lab partners [16] to discuss experimental and computational results for projects focused on developing membranes for high temperatures PEMFCs. To address the challenges to developing improved membranes the DOE has funded a number of projects focusing on high-temperature membrane R&D as part of the HTMWG [18,19,20,21,22,23,24,25,26,27,28,29] and prior to forming the HTMWG [30]. Several different strategies have been investigated. One strategy was to control polymer chemistry to increase the local concentration of proton conducting groups while maintaining mechanical properties, and to allow for stable operation at low RH [18,20,26,27,29,30]. Another strategy was to employ anhydrous proton conductors to provide a water-independent proton conducting mechanism [20,23,25,26,28]. Also, separation of the ion conducting function from the structural support function was pursued through the development of composite membranes [19,21,23]. Many of the projects highlighted have elements of more than one strategy, so the strategies should not be taken as mutually exclusive avenues of development.

**Figure 2:**
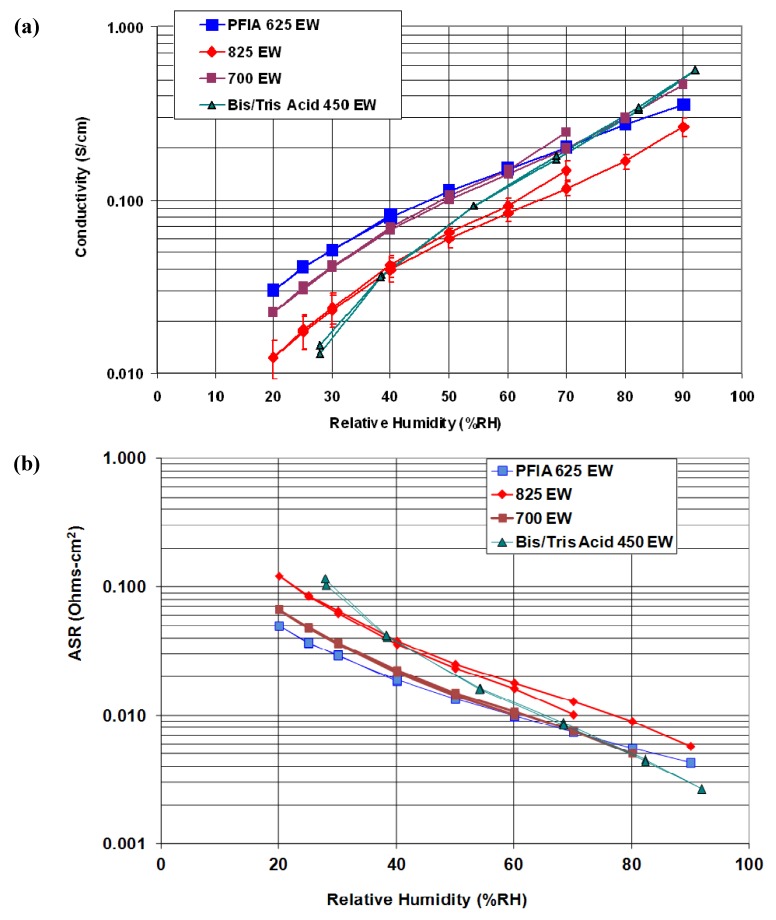
Conductivity of several 3M membranes at 80 °C over a range of relative humidity (RH) showing that low EW perfluoroimide acid (PFIA) has similar conductivity as 700 EW PFSA [31]. (**a**) The experimentally measured conductivity; (**b**) the calculated ASR for 15 micron membranes.

**Figure 3:**
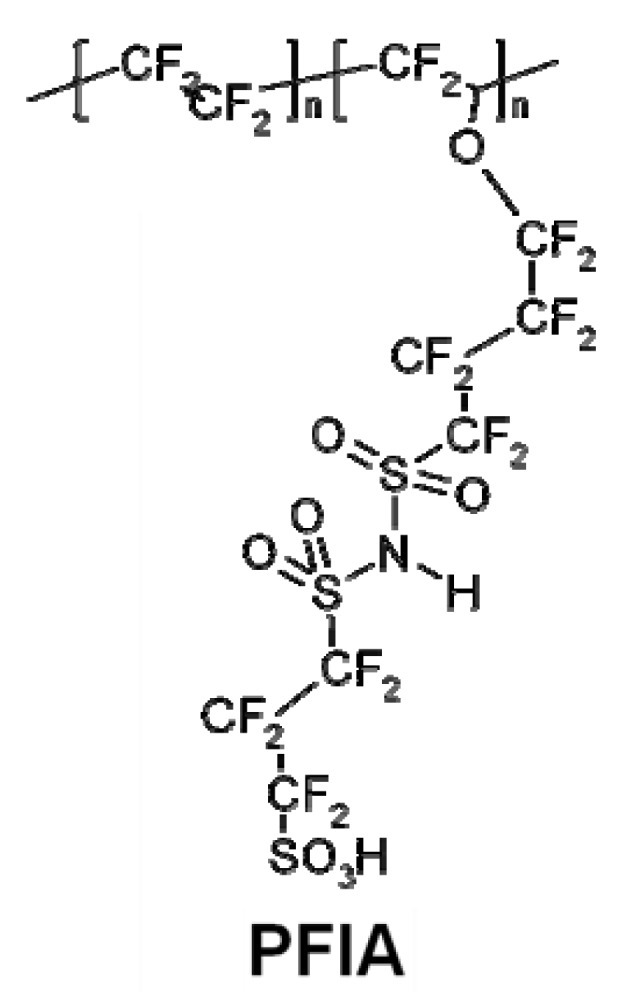
Structure of the 3M low EW perfluoroimide acid (PFIA) [31].

One approach to achieving low equivalent weight without sacrificing mechanical properties involves the incorporation of multiple proton donors per side chain. This approach has been investigated at 3M using PFSA-type polymers, based on 3M’s existing ionomer chemistry, which were modified to include multiple acid sites on the polymer side chain while maintaining the same TFE backbone [9,17,30,31]. The existing 3M ionomer contains 4-carbon side sulfonated side chains attached to the TFE backbone through an ether linkage. 3M had previously investigated reduction in EW through increasing the density of side chains, but while the low EW polymers performed well at low RH, the low backbone crystallinity and high water uptake of these polymers resulted in poor mechanical properties and high water solubility at elevated temperatures. To bypass this shortcoming, 3M developed ionomers with multiple acid groups per side chain, thus providing a high density of acid groups while maintaining the long TFE backbone segments required for crystallinity [9,31]. 3M investigated several approaches to introduce multiple acid groups per side chain, but achieved the best results by incorporating a superacid bis sulfonyl imide group within the side chain [17]. The imide group is an even stronger acid than the sulfonic acid group. 3M has used the imide as a protogenic group and linking moiety to prepare several multi-acid side-chain ionomers, allowing the conductivity to remain high under drier conditions. Using this approach, 3M was able to utilize the same backbone as a 1000 EW ionomer, but obtain an EW of ~640. A variety of different multiacid polymers were investigated. The best performing multi-acid ionomer, as shown in Figure 2, was the 625 EW perfluoroimide acid (PFIA) depicted in Figure 3, prepared from an 825 EW PFSA backbone material. The 625 EW PFIA was down-selected for final analysis. 

Mechanical stability of the PFIA ionomer was improved using a chemically inert nanofiber support which reduced linear swelling in water by as much as 15-fold [9,31]. The supported and chemically stabilized PFIA membrane was subjected to the DOE membrane durability tests, where it exceeded 20,000 RH cycles in the mechanical durability test and lasted over 600 h at OCV in the chemical durability test, exceeding the DOE targets. The 15 micron thick supported PFIA membrane also met the ASR targets at −30, 20, and 80 °C and decreased the ASR at 120 °C and P_H2O_ of 40 kPa to 0.023 Ohm cm^2^ (corresponding to a conductivity of 0.087 S/cm), just slightly above the target of 0.02 Ohm cm^2^ [31]. The conductivity under less challenging conditions (0.3 S/cm at 80 °C, 95% RH) is greatly improved over traditional PFSA membranes, which should lead to significant improvements in performance. Tests of PFIA membrane incorporated into a membrane electrode assembly provide indirect evidence that PFIA also meets the DOE crossover and electrical resistance targets. While all of the projects described in this review have made progress towards meeting all membrane targets, PFIA has made the greatest progress in meeting all the targets simultaneously. Going forward, 3M intends to build on this technology to gain further understanding of the factors influencing conductivity and durability in this membrane and develop new materials based on this understanding [31].

**Figure 4:**
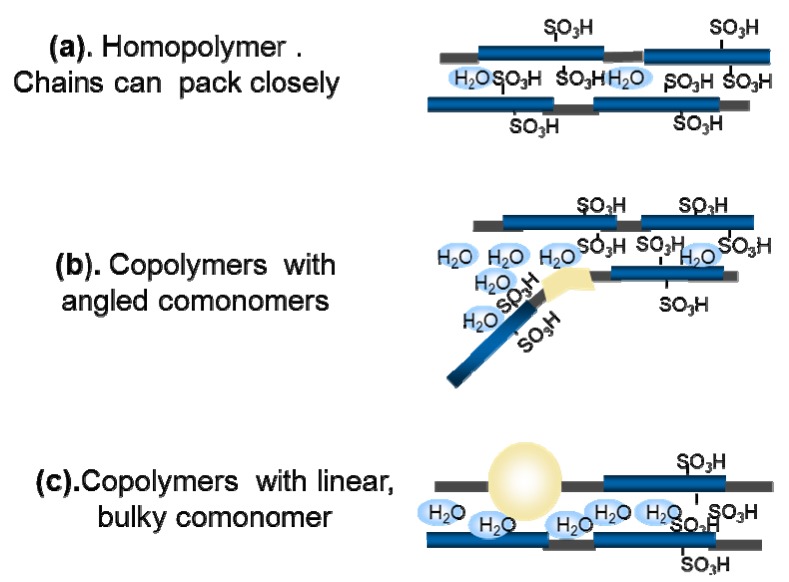
Schematic representation of the approach taken at the Case Western Reserve University. In (**a**) the unmodified polymers pack close together; (**b**) shows the effect of adding angled copolymers which forces the chains apart over the whole length creating permanent nano-pores lined with sulfonic acid groups; and (**c**) shows the effect of adding bulky monomers creating nano-pores lined with sulfonic acid groups [32,33].

Another approach to provide high conductivity under hot and dry conditions involves the use of rigid rod hydrocarbon polymers containing nanopores lined with sulfonic acid groups. These hydrophilic pores promote water condensation, yielding a high concentration of mobile protons at low RH [32,33]. Hydrocarbon membranes are of interest for PEMFC applications [33], particularly for early-market applications, since the cost of hydrocarbon ionomers is significantly lower than the cost of perfluorinated ionomers when the materials are produced at low volume [5]. In Figure 4, a schematic of the concept as developed at CWRU is shown. 

Poly(phenylene sulfonic acid) has a small cross-section backbone with projecting sulfonic acid groups with absorbed water separating the chains [32,33]. Adding angled or bulky co-monomers forces the chains apart over the whole length, creating permanent nanopores lined with SO_3_H groups that should tightly bind water. Since the channels are lined with sulfonic acid groups, they provide high-conductivity pathways for protons. The effect on conductivity is much greater for poly(phenylene disulfonic acid) (PPDSA) polymers than for poly(biphenylene disulfonic acid) (PBDSA) polymers, about an order of magnitude higher for PPDSA between 25 and 75 °C. The polymer can be dimensionally stabilized by grafting cross-linking groups on the backbone and subsequently crosslinking them. Grafted groups protrude further from the backbone than the acid groups; this increases the chain separation and thus can increase the frozen-in free volume. This structural design generates non-collapsible nanopores lined with a high density of sulfonic acid groups that hold water very strongly. PPDSA membranes have excellent ASR at 120 °C and 40 kPa water partial pressure (<0.01 Ohm cm^2^). The PPDSA membranes were observed to have low electrical resistance (31 Ohm cm^2^) under standard testing protocols; however, this may be due to cracks forming in the membrane during MEA preparation [33], thus mechanical properties may need to be improved. Progress towards improving the membrane brittleness was demonstrated by modifying the MEA preparation and pressing a catalyst coated gas diffusion layer onto the membrane. Samples made using the modified MEA fabrication process were tested for 11 days at 35% RH over a range of temperatures up to 95 °C with no gas permeation; testing at 100% RH caused the film to tear [34]. The CWRU group devoted considerable effort to understand the impact of copolymer content and crosslinking on mechanical stability. They found that swelling in the polymer may be due to inhomogeneity in the crosslink distribution and that crosslinking reduced proton conductivity by as much as 20% for membranes over a range of graft concentration of 7%–12%. The CWRU team found that grafting using biphenyl sulfone under dry nitrogen improved the homogeneity. 

**Figure 5:**
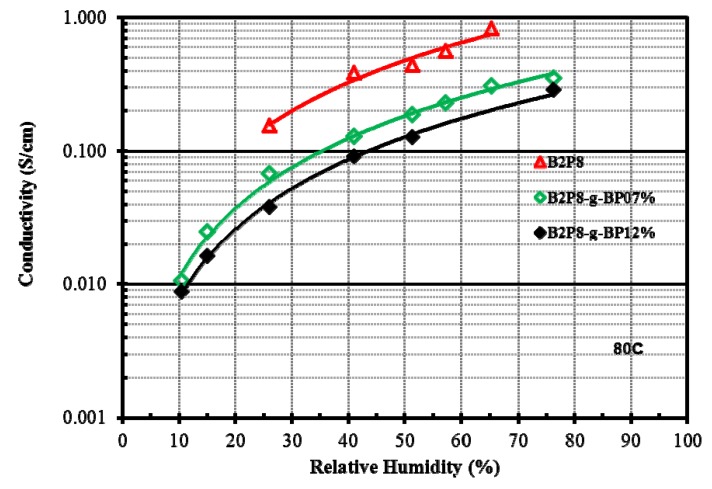
Conductivity as a function of RH measurements of best-in-class biphenyl-PPDSA with cross-linked polymers measured at 80 °C [32,33]. Here PPDSA is poly(phenylene disulfonic acid.

Conductivity curves over a range of RH for the best performing CWRU membranes are shown in Figure 5. Another strategy to improve mechanical strength was to incorporate carbon nanotubes (CNTs) into the membrane. It was found that up to 3% (w/w) CNTs could be added without substantially altering the electrical conductivity of the membrane [34]. Two approaches have been suggested to address durability of the frozen-in free volume: increasing the molecular weight, and improving the grafting chemistry [34]. Molecular weight is limited by polymer precipitation as the reaction proceeds. A method to increase polymer solubility during polymerization and thus increase chain length has been proposed by polymerizing at 200 °C, which was found to give higher molecular weight polymers. Very reactive moieties that can graft homogeneously on PPDSA using a common solvent that is sufficiently inert to allow grafting of such groups should improve mechanical properties and stability. One co-monomer, 2,7-dibromofluorene 3,5-disulfonic acid, has shown promise; it copolymerizes randomly and could be post-grafted. Finally, the polymers can be put into an expanded matrix of, or co-cast with a reinforcing material to improve mechanical properties.

Another strategy focused primarily on developing an anhydrous approach to provide acidic protons through the use of alternative proton conductors such as heteropoly acids [35,36], tethered phosphonic acids [20], and imidazoles [24]. Heteropoly acids (HPAs) have high proton conductivities, synthetic versatility, and are known to decompose peroxides [35,36]. The approach taken by the Colorado School of Mines, in partnership with 3M, was to functionalize HPAs with excellent inherent conductivity, such as K_8_[SiW_11_O_39_]∙13H_2_O, with monomers so that they could be fabricated into PEMs with the use of a suitable co-monomer. Acrylates were initially chosen as the co-monomers because acrylates represent a well-known, readily accessible polymer system, leaving the synthetic effort to be devoted to making the HPA monomers. Acrylate-based polymer systems allowed the chemistry to be easily varied so that the effect of morphology could be studied. Acrylate-based HPA membranes were prepared with proton conductivity exceeding 0.1 S/cm at 120 °C and 50% RH [36].

**Figure 6:**
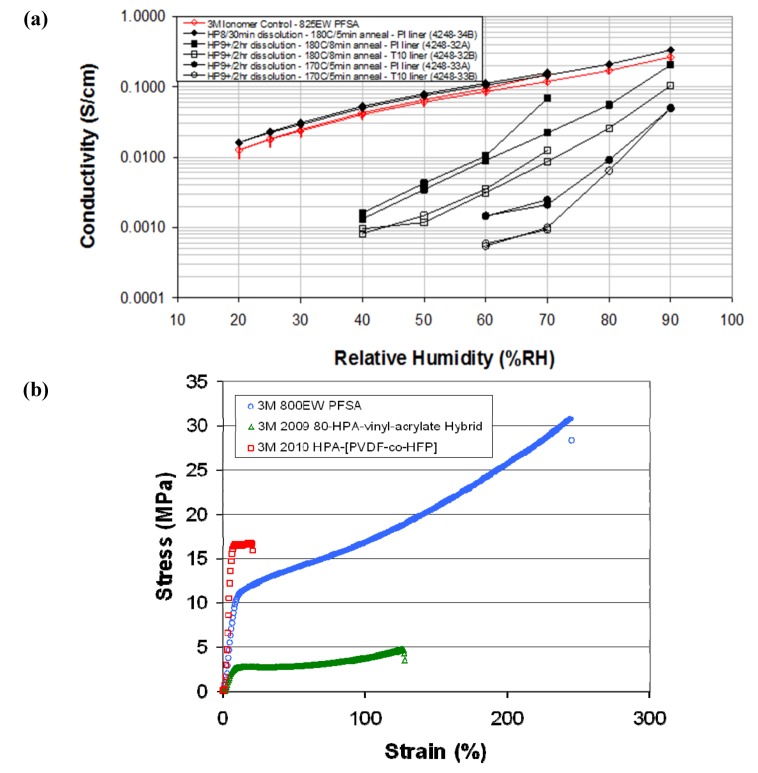
Colorado School of Mines HPA membrane. (**a**) Conductivity data for the HPA functionalized Dyneon™ polymer; (**b**) stress strain curves comparing the HPA Dyneon™ polymer, the 3M 825 PFSA ionomer, and an acrylate polymer [35,36].

However, acrylates are inherently unsuitable for fuel cell membranes due to the instability of the ester linkage. After demonstrating the feasibility of HPA membranes with the acrylate system, more suitable polymer systems were investigated. Two perfluorinated polymers were selected for their chemical and mechanical stability, trifluorovinyl ether (TVFE) and Dyneon™. The general approach to attaching HPAs to perfluorinated backbones was either through silane linkages to functionalized TVFE or by attachment to phosphonated Dyneon™. Films formed from the HPA functionalized Dyneon™ polymer show excellent mechanical properties and ion conductivity when compared to 825 EW PFSA as shown in Figure 6. However, there was some loss of HPA and conductivity upon leaching. The authors suggested that all DOE targets could be met if the HPA loading and film properties of the polymer were optimized. The two key challenges that need to be addressed are utilization of all protons under elevated temperatures, dry conditions, and immobilization of the water-soluble HPA [36].

The use of chemically inert mechanical supports to strengthen and stabilize membrane ionomers is a promising approach to achieve good membrane mechanical properties with low equivalent weight ionomers. For instance, in a project led by Giner, dimensionally stable membranes (DSMs) were formed using support materials, such as polysulfone and polyimide (Kapton), that were impregnated with commercially-available PFSAs and novel ionomers [37,38]. Two-dimensional (2DSM) and three-dimensional (3DSM) supports were developed and tested to stabilize thin membranes of low equivalent weight PFSAs. The 2DSM approach was initially prepared using laser-drilling of a continuous film of the support polymer, followed by penetration and encapsulation by PFSA, though lower-cost techniques have recently been developed (see below). The 3DSM approach used impregnation methods to introduce PFSA into commercially available porous supports (Millipore). Both 2DSM and 3DSM, result in significantly improved *x*-*y* dimensional stability. Laser-drilled 2DSM membranes reduce *x*-*y* swelling to less than 5% [37], and 3DSM membranes are reported to have the same dimensional stability improvements as the 2DSM [39]. Each approach poses unique challenges. Complete filling of the 3DSM pores with PFSA initially proved to be challenging while incorporating PFSA into 2DSM does not present a significant challenge. On the other hand, 2DSM poses a challenge to scalable manufacturing with processes such as laser drilling being cost-prohibitive. 

**Figure 7:**
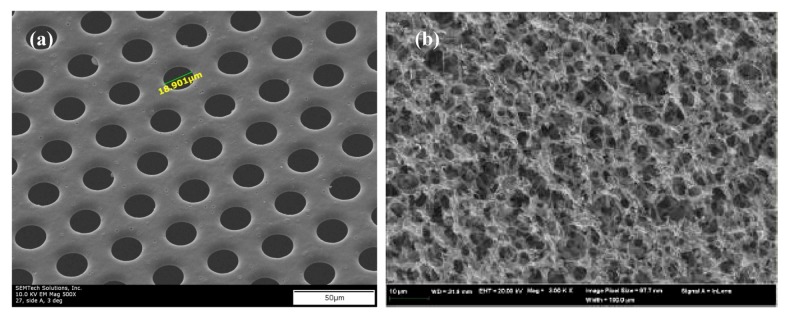
Micrographs of dimensionally stable membranes (**a**) Two-dimensional stable membranes (2DSM); (**b**) three-dimensional stable membranes (3DSM) [38,39].

**Figure 8:**
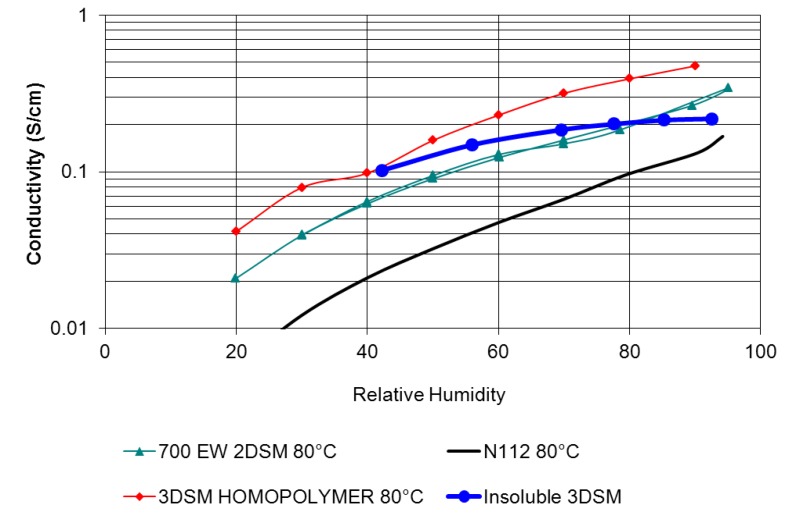
Comparison of conductivity between Giner’s PFSA-filled 2DSM over a range of relative humidity (RH) *vs.* Nafion 112^®^ [38].

In Figure 7 SEM images of both the 2DSM and 3DSM are shown. The DSM shown in Figure 8 significantly outperforms Nafion 112^®^, except at 30 °C above 80% RH, where the performance is similar. Conductivity of the composite membrane scales with the volume fraction of PFSA ionomer. By tuning the amount of support material, researchers at Giner were able to nearly eliminate *x*-*y* swelling. For example, 2DSM composite membranes composed of 10% support with no greater than 60% void space demonstrated <5% with conductivity of approximately 90% of the pure ionomer conductivity [37]. For 3DSM composite membranes, they demonstrated similar reductions in *x*-*y* swelling while making gains in conductivity [37]. As a consequence of eliminating swelling, the membrane is stable during freeze-thaw cycling and RH cycling during normal fuel cell operations. Though 2–3 times more conductive than Nafion^®^ 112, DSM membranes have yet to demonstrate achievement of the DOE ASR targets. To close the gap between the demonstrated DSM conductivity and the DOE ASR targets, PFSAs with even higher acid content have been synthesized, using cross-linking to provide polymer insolubility. Giner is also developing scalable processes for preparing supports that will result in even thinner membranes (~15 µm) with lower manufacturing cost. Composite membranes are approximately 2× the thickness of the support material, thus an approximately 8 µm support is needed to form a 15 µm composite membrane. Giner has successfully formed UV-cured thiolene, mechanically deformed polysulfone and phase-inverted solvent cast polysulfone membranes that are 7–8 µm thick. Mechanical deformation of polysulfone is the best scalable route with proven materials cost of $50/m^2^ for batch process and is anticipated to be lower for roll-to-roll. Work remains to increase the hole size for mechanically deformed membranes. Open area target is 50%; the current mechanical processes result in films with 15%–30% open area. DSMs show promise to be inexpensive and scalable, but the cost of PFSA remains as a challenge [38,40]. Giner has demonstrated three viable pathways targeting cost of $20/m^2^ or less, including UV micro-replication, mechanical deformation, and inversion casting methods.

**Figure 9:**
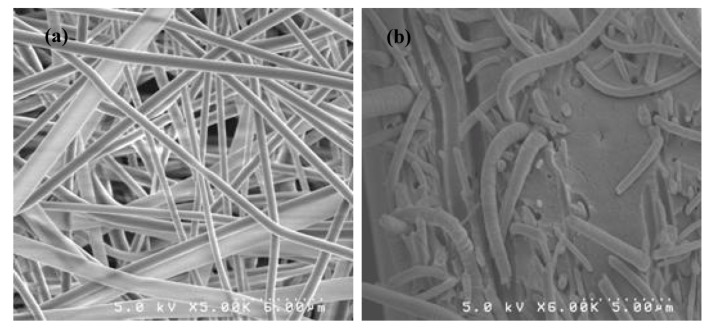
Scanning electron micrographs of electrospun nanofiber composite membranes prepared at Vanderbilt University. (**a**) Top down view of a dual nanofiber mat composed of Nafion and polyphenylsulfone nanofibers; (**b**) Freeze-fractured cross section of a membrane where Nafion perfluorosulfonic acid polymer is surrounding nanofibers of polyphenylsulfone.

A similar approach involves the use of inert nanofiber mats of engineering polymers, such as polyphenylsulfone (PPSU), to serve as mechanical stabilizers for low equivalent weight ionomers that permeate the mat, as well as the inverse approach, in which mats of ionomer nanofibers are permeated with engineering polymers. Such membranes were prepared at Vanderbilt University by a newly developed dual nanofiber electrospinning technique to produce a mat containing PFSA and PPSU [41,42]. Membranes were made where: (i) PFSA nanofibers were surrounded by a PPSU matrix by a treatment where exposure of the dual-fiber mat to solvent vapors causes the PPSU to flow; (ii) PPSU nanofibers were surrounded by PFSA ionomer by annealing, causing the PFSA to flow forming a structure in which the PFSA fills the voids in a 3D network of high-strength, high-stability nanofibers. Scanning electron micrographs of dual nanofiber mats are shown in Figure 9. 

**Figure 10:**
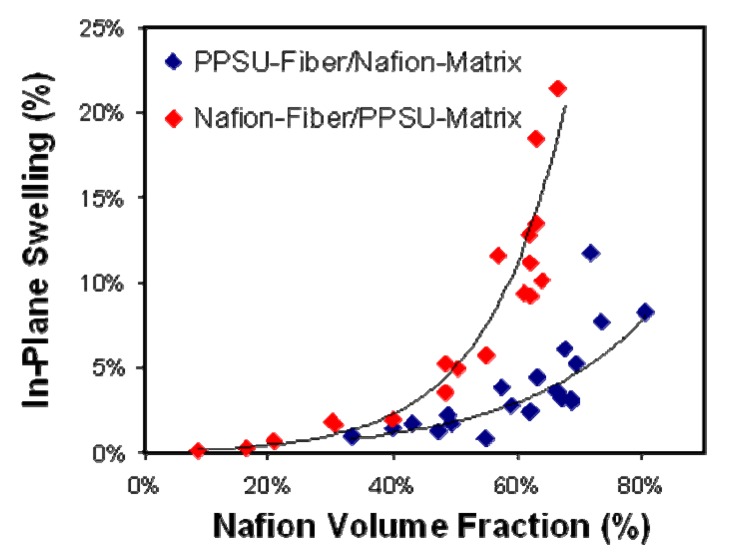
Comparison of Vanderbilt University’s in-plane swelling for polyphenylsulfone (PPSU)/Nafion and Nafion/PPSU nanofiber membranes in 100 °C water as a function of Nafion volume fraction [42].

Membranes of these types experience lower swelling in liquid water than pure PFSA membranes, and greater durability in RH cycling. Membranes were evaluated and compared in terms of water uptake, in-plane proton conductivity, volumetric and in-plane swelling in water, and mechanical properties. The proton conductivity and volumetric/gravimetric water swelling were found to be identical for the two membrane structures. Pintauro *et al.* found that volume swelling of PPSU/Nafion nanofiber composites is controlled by the volume fraction of PPSU and is lower than predicted by the linear mixing rule [43]. In-plane swelling of Nafion after soaking in 100 °C water is 37%. In-plane swelling of composite nanofiber membranes is significantly lower than Nafion as shown in Figure 10. The membrane composed of a Nafion matrix with embedded PPSU fibers demonstrated restricted in-plane swelling less than 5% for 60% Nafion content, while the Nafion fiber/PPSU matrix swelled <15% at 60% Nafion content, *versus* a predicted 22% by the linear mixing rule. Dual fiber composite membranes were prepared and evaluated in an MEA [42], where a PPSU nanofiber mat (30 vol %) was surrounded by Nafion (70 vol %) and performance and durability compared to a Nafion 212 based MEA. The composite membrane had a thickness of 36 µm, an areal resistance of 45 mΩ cm^2^ and in-plane swelling of 6% while the Nafion 212 membrane had a thickness of 51 µm, areal resistance of 45 mΩ cm^2^, and in-plane swelling of 37%. Beginning of life performance at 80 °C and 100% RH was indistinguishable. In-plane water swelling was 5% in water and the conductivity was 0.093 S/cm at 120 °C at 50% RH. The membrane was fabricated into a fuel cell MEA by collaborators at 3M and tested at low RH in H_2_/air fuel cell. The MEAs were subjected to a combined chemical/mechanical durability test where the MEA was held at OCV in H_2_/air cycling between 100% RH (2 min) and 0% RH (2 min). The membrane performed significantly better than Nafion 212 for 25% < RH < 93% and T = 100 °C and 120 °C. The composite membrane MEA had a 54% increase in lifetime (as measured in time for OCV to drop below 0.8 V) compared to the Nafion 212 MEA. 

**Figure 11:**
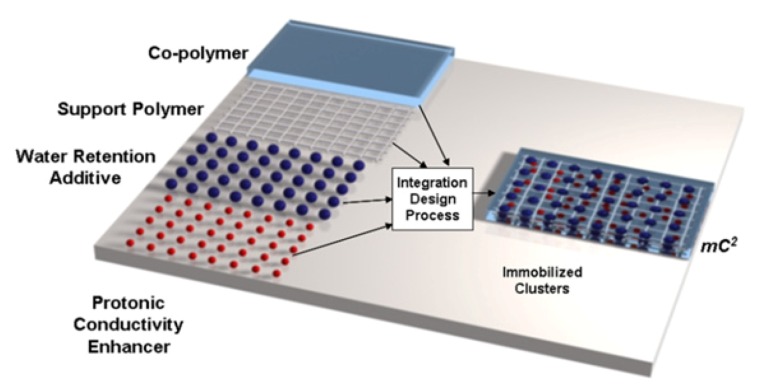
Schematic of the multi-component composite membrane (mC2) by FuelCell Energy [44].

**Figure 12:**
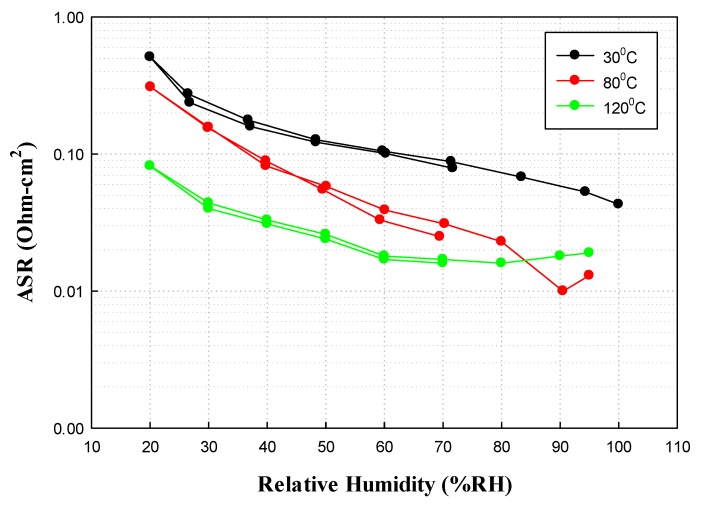
Through-plane area specific resistance (ASR) for the mC2 membranes measured at 30, 80, and 120 °C showing an average ASR of 0.025 Ohm cm^2^ at 50% RH and 120 °C compared to the DOE target of <0.02 Ohm cm^2^.

In addition to the use of composited materials as mechanical stabilizers, they may also serve as conductivity enhancers. Additives are under investigation for application as water-retaining agents and as proton-donating agents. For instance, a multi-component composite membrane (mC2) developed at FuelCell Energy is composed of three functional materials, namely: an ionomer; a water retention additive; and a protonic conductivity enhancer. In particular, the composite membranes are formed from a short side-chain, low EW PFSA with increased molecular weight, good mechanical properties and proton conductivity; a zeolite nanoparticle additive with high water uptake capacity without dimensional change, which enhances water retention at the low RH conditions and enhances the composite membrane’s proton conductivity by providing an alternate conduction path; and a superacid, which increases the density of mobile protons at all operating conditions [44,45,46]. Figure 11 shows a schematic of the composite membrane. Membrane electrode assemblies are being optimized and tested. The mC2 membranes have shown pathways to meet DOE targets for hydrogen crossover, ASR at 80 °C and less than 45 kPa water partial pressure, electrical resistance, and performance at rated power. Addition of the nano-zeolite and superacid increased conductivities by more than 70% over the baseline PFSA and proposed that the additives provide alternate proton conducting pathways. Good progress towards meeting the DOE ASR targets at 120 °C and 40 kPa water partial pressure and performance at 0.8 V has also been demonstrated [46]. The membrane conductivity at 120 °C and 50% RH is 0.113 S/cm, and the membrane ASR under these conditions is 0.025 Ω cm^2^. However, the ASR increases to ~0.08 Ohm cm^2^ at 120 °C and 20% RH as shown in Figure 12. Durability remains to be evaluated. R&D costs suggest a pathway to meeting the $20/m^2^ cost target. In addition to targeting transportation fuel cells, FuelCell Energy is considering use of these membranes for co-production of hydrogen from high-temperature fuel cells. 

### 3.2. DMFC Membranes for Portable Power Applications

Challenges for DMFC systems include reducing methanol crossover to increase performance and efficiency, and simplifying the balance of plant, to increase energy and power density, improve reliability, and reduce cost. To overcome system level challenges, DOE has set targets addressing, specific power, power density, cost, and durability for a range of system power levels (<2 W, 10–50 W, 100–250 W) [3]. The major technical challenges for DMFC membranes are to reduce methanol crossover and to manage water in the membrane and at the anode. In addition to the targets listed in Table 1, the DOE has also set critical milestones for developing DMFC membranes with methanol permeability less than 1 × 10^−8^ cm^2^/s by 2015. Current membrane efforts at the DOE focus on reducing methanol crossover of PFSA type membranes and hydrocarbon based block copolymer ionomers. Water management is also being addressed, but is beyond the scope of this paper. 

**Figure 13:**
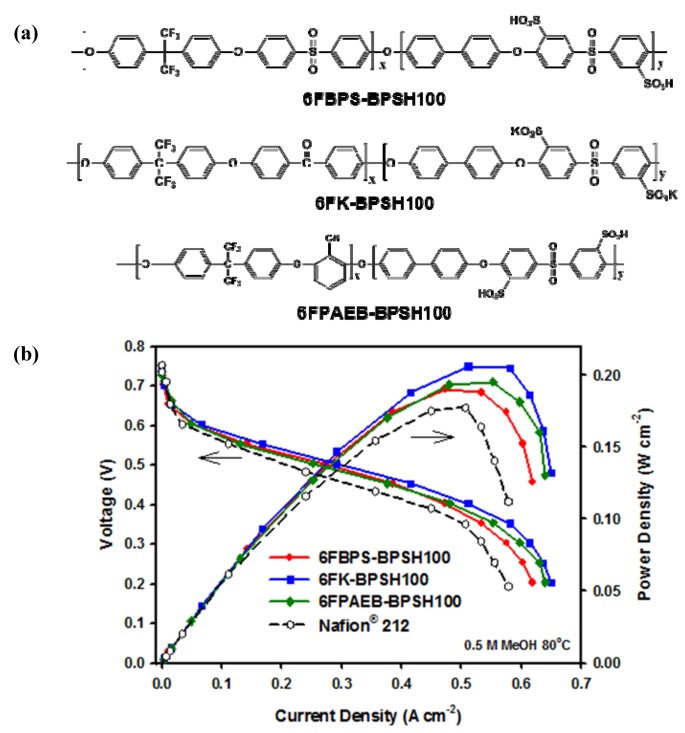
(**a**) Multiblock copolymers for DMFC membranes with reduced crossover; (**b**) Comparison of fuel cell performance for several multiblock copolymers shown in (**a**) compared to Nafion 212^®^ demonstrating improved performance as the 6F:BP ratio is tuned [48].

One pathway to decrease methanol permeability involves the use of multiblock copolymers [47] as membrane ionomers. In a comprehensive DMFC for portable power project led by Los Alamos National Laboratory, researchers at Virginia Tech have demonstrated the applicability of multiblock copolymers in DMFC membranes [48,49]. Partial fluorination of a hydrophobic block was used to enhance proton conductivity via better phase separation and to improve adhesion to the Nafion^®^ ionomer used in the electrodes. Recently, disulfonated poly(arylene ether sulfone) (BP) and hexafluorinated (6F) bisphenol were employed as block copolymers with improved phase separation. Methanol permeability was reduced by the addition of functionality on the 6F (e.g., the nitrile on 6FPAEB) via a complexation reaction of methanol with water [48]. By optimizing the ratio of BP to 6F, the methanol crossover has been decreased by 55% (0.08 A/cm^2^* vs.* 0.18 A/cm^2^ crossover for Nafion^®^), while meeting fuel cell current density targets [50]. In Figure 13, several 6Fx-BPy multiblock copolymers are shown along with a power curve for the best performing membrane.

The improved multiblock copolymer allows for: (i) current density > 0.28 A/cm^2^ at 0.5 V; (ii) methanol utilization of >95% at peak power; and (iii) less than 10% DMFC performance degradation for 100 h in a preliminary life-test at 80 °C and 0.5 M methanol by reducing methanol crossover by 55% relative to Nafion^®^ 212 and by 40% relative to the best earlier multiblock copolymers. While DMFC performance strongly depends on methanol concentration, the unrecoverable performance loss with 0.5 M MeOH feed is relatively small; durability improvements in the presence of higher methanol concentrations are currently being addressed.

**Figure 14:**
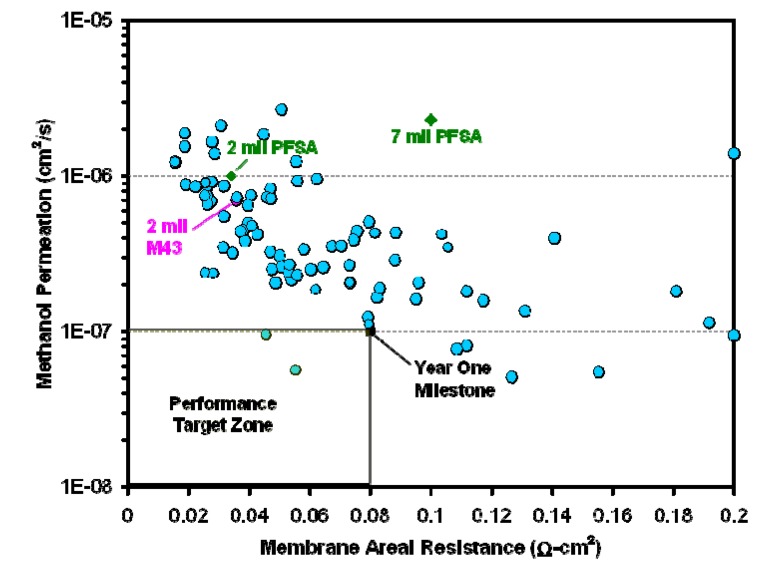
Results of DMFC membrane screening for areal resistance and methanol permeability [52].

An alternative approach to reducing methanol permeability involves the use of composite membranes, in which a non-conductive phase serves to block methanol crossover. This approach is being pursued at Arkema using Kynar^®^ poly(vinylidene fluoride) (PVDF) polymer blends which are chemically stabile and mechanically strong. Due to PVDF’s impermeability to methanol and its compatibility with a number of polyelectrolytes, composite membrane compositions can be tailored to minimize methanol permeability while optimizing conductivity and mechanical properties. More than 100 composite membranes composed of a sulfonated and phosphonated hydrocarbon polyelectrolyte developed in an earlier project [49], PVDF, and cross-linking agents [51] were screened for methanol permeability and areal resistance. Results of the screening tests are shown in Figure 14. 

While testing of the composite membranes focused on *ex situ* conductivity and methanol permeability, additional testing such as microscopy, mechanical testing and crystallinity measurements were carried out on a limited number of samples to understand the structure-property relationships and morphology. Arkema demonstrated progress towards meeting the project milestones of areal resistance ≤0.08 Ohm cm^2^ and a permeability coefficient of ≤5 × 10^−8^ cm^2^/s by December 2012 and have scaled up membrane processing on a pilot line to demonstrate scalability of the process. The best performing membranes currently have 0.08 Ohm cm^2^ ASR and methanol permeability of 8 × 10^−8^ cm^2^/s as of May 2012 for membrane thicknesses of 0.6–0.9 mils [52]. Membranes were incorporated into full MEAs with electrodes made with PFSA binders. Adhesion between the PFSA binder and PVDF is good due to the partially fluorinated PVDF matrix. Arkema found that MEA performance is largely determined by MEA ASR at low methanol concentration (<3 M), a regime in which the Arkema MEAs performed similarly to 2–7 mil PFSA MEAs. However, at high methanol concentrations (>5 M), the Arkema membranes outperform PFSA, due to significantly lower methanol crossover. Most MEAs tested failed between 500 and 1000 h due to performance losses. The losses were attributed to the electrodes, but higher areal resistance and lower methanol crossover were observed to develop over time, indicating that further study is needed to understand the underlying mechanism. Recently, efforts have been under way to develop and incorporate sulfonated silica particles into the membranes to improve conductivity. Conductivity and methanol permeability both decrease on addition of 3-trihydroxysilyl-1-propane sulfonic acid (TPS) to the membranes, while selectivity is improved. Further testing is needed to understand the effects of TPS additives. 

## 4. Conclusions

The Department of Energy Fuel Cell Technologies Program maintains a portfolio of innovative R&D projects working to develop fuel cell technologies for wide spread commercial applications. Developing membranes for PEMFCs is a key component of the portfolio. DOE-supported membrane R&D has enabled the development of membranes that meet most of the DOE technical targets. PFIA meets all but the most aggressive membrane targets for 2017 and many of the other membrane projects show good progress towards meeting the targets. Because membranes are among the costliest components at low volume manufacturing, advances could have a major impact on how rapidly FCEVs penetrate the market. Work on many of the projects discussed continues to address the remaining challenges, including meeting the targets for ASR at the hottest, driest conditions, while new projects are addressing challenges associated with high volume manufacturing and optimizing membranes incorporated into membrane electrode assemblies. 
